# Transcriptome Analysis of the Human Tibial Nerve Identifies Sexually Dimorphic Expression of Genes Involved in Pain, Inflammation, and Neuro-Immunity

**DOI:** 10.3389/fnmol.2019.00037

**Published:** 2019-03-05

**Authors:** Pradipta R. Ray, Jawad Khan, Andi Wangzhou, Diana Tavares-Ferreira, Armen N. Akopian, Gregory Dussor, Theodore J. Price

**Affiliations:** ^1^School of Behavioral and Brain Sciences, The University of Texas at Dallas, Richardson, TX, United States; ^2^Center for Advanced Pain Studies, The University of Texas at Dallas, Richardson, TX, United States; ^3^Department of Endodontics, University of Texas Health San Antonio, San Antonio, TX, United States

**Keywords:** sex-differential gene expression, human peripheral nerve transcriptome, peripheral nervous system sex differences, pain genes, pro-inflammatory genes

## Abstract

Sex differences in gene expression are important contributors to normal physiology and mechanisms of disease. This is increasingly apparent in understanding and potentially treating chronic pain where molecular mechanisms driving sex differences in neuronal plasticity are giving new insight into why certain chronic pain disorders preferentially affect women vs. men. Large transcriptomic resources are now available and can be used to mine for sex differences to gather insight from molecular profiles using donor cohorts. We performed in-depth analysis of 248 human tibial nerve (hTN) transcriptomes from the GTEx Consortium project to gain insight into sex-dependent gene expression in the peripheral nervous system (PNS). We discover 149 genes with sex differential gene expression. Many of the more abundant genes in men are associated with inflammation and appear to be primarily expressed by glia or immune cells, with some genes downstream of Notch signaling. In women, we find the differentially expressed transcription factor SP4 that is known to drive a regulatory program, and may impact sex differences in PNS physiology. Many of these 149 differentially expressed (DE) genes have some previous association with chronic pain but few of them have been explored thoroughly. Additionally, using clinical data in the GTEx database, we identify a subset of DE, sexually dimorphic genes in diseases associated with chronic pain: arthritis and Type II diabetes. Our work creates a unique resource that identifies sexually dimorphic gene expression in the human PNS with implications for discovery of sex-specific pain mechanisms.

## Introduction

Sex-differential gene regulation and resultant changes in transcriptome, proteome, and metabolome shape sexually dimorphic physiology and behavior in animals. Sex-differential molecular profiles in human tissues have a profound effect on health, resulting in disease susceptibility, prevalence and pathophysiology differences between sexes. Acute and chronic pain have a staggering global disease burden, and prevalence of many chronic pain conditions like fibromyalgia and neuralgia have been shown to be higher in women (De Toledo et al., 2016), and sex-differential molecular changes in the peripheral and central nervous system (CNS) have been implicated in preclinical models (Mogil, 2012). Transcriptome profiles of human Dorsal Root Ganglia (DRG) and Trigeminal Ganglia have been characterized previously (Flegel et al., 2015; LaPaglia et al., 2018; Ray et al., 2018) but these studies are underpowered for capturing subtle transcriptional changes between sexes. Human tibial nerve (hTN) transcriptomes, which contains axons from DRG neurons, along with a panel of other harvested tissues, have been profiled using RNA-sequencing (RNA-seq) in hundreds of male and female donors as part of the GTEx project (Lonsdale et al., 2013). Some studies have characterized sex-differential gene expression changes (Chen et al., 2016; Gershoni and Pietrokovski, 2017) and investigated the evolutionary and regulatory basis of such changes across the repertoire of GTEx tissues, but none have focused on the hTN.

We cataloged sex differences in the hTN transcriptome under baseline conditions focusing on potential functional impact in the context of pain, inflammation and neuro-immunity. Previous analysis of the human peripheral nerve transcriptome has focused on changes in disease pathologies like nerve sheath tumors (Lee et al., 2014), or diabetic neuropathy (Hur et al., 2011; Luo et al., 2017). To our knowledge, sexual dimorphism studies on mammalian peripheral nerves is limited to a single microarray study in a rodent model of Type II diabetes (O'Brien et al., 2016). Our work thus fills an important gap in transcriptome studies of the human PNS and we created an online database (https://www.utdallas.edu/bbs/painneurosciencelab/sensoryomics/sexdiffnerve/) identifying sex differentially expressed genes in the hTN, and identified potentially functionally important genes by intersectional analysis with relevant databases. We further analyzed the cell type of expression of these genes, putatively identifying axonally localized transcripts. We also investigated the possible regulatory role of sex hormones in the sexual dimorphic genes by analyzing correlation of age with gene expression.

## Materials and Methods

### Data Use Policy

We only analyzed anonymized samples for which the corresponding donor consent information was available in the GTEx dataset (dbGAP phs000424.v7.p2) at the time of analysis. All of the datasets we analyzed were approved for General Research Use (GRU) and thus have no further limitations outside of those in the NIH model Data Use Certification Agreement. Our work was further approved by the UT Dallas IRB (protocol number 15-237).

### Donor Selection by GTEx Consortium

The Genotype Tissue Expression (GTEx) consortium performed RNA-seq on a panel of harvested tissues, including the hTN, in a large cohort of consented tissue, organ or post-mortem donors. Donors of both sexes, from all ancestry groups between the ages of 21–70, and with Body Mass Index (BMI) between 18.5 and 35 were eligible. Post-mortem donors were also constrained to have had no whole blood transfusion within 48 h prior to death, and time between death and tissue collection was constrained to be <24 h. Donors were further restricted to have no history of metastatic cancer, no chemotherapy or radiation therapy within 2 years prior to death, and no communicable diseases that disqualify people for organ or tissue donation.

### Sample Processing by GTEx Consortium

This provided a diverse dataset that is uniquely suitable for studying differences across sub-cohorts, such as sex differences in our study. Post-excision, aliquots were stabilized in a solution containing ethanol and methanol, acetic acid and Paxgene Tissue fixative (Qiagen). A portion of each tissue was then subject to RNA extraction and quantification. Next Generation sequencing (RNA-seq) was performed using the Illumina TruSeq platform, averaging ~50 million reads per sample. Sequenced reads were mapped to the human reference genome and transcriptome using the STAR toolkit (Dobin et al., 2013) and relative abundance quantified as Transcripts per Million (TPM) using the RSEM tool (Li and Dewey, 2011). RNA quality was checked using the RNA Integrity Number, and sequencing quality was analyzed using the RNA-SeqC toolkit (DeLuca et al., 2012). Donor level data included basic demographics, use of medication, medical history, results of laboratory tests, and circumstances of death were collected from the donor or next of kin and compared with the medical record. Donor level data and sample level data (which includes ischemic time, and comments from prosector and pathology reviewer) were analyzed for completeness and quality before release by the consortium. Further details of bench protocols and computational pipelines used by the GTEx consortium can be obtained from Lonsdale et al. (2013).

### GTEx Data Requantification

PAXgene preserved hTN RNA-seq samples (dbGAP phs000424.v7.p2) with total RNA sequenced on the Illumina Truseq platform (and available donor consent information at the time of analysis) were identified. Samples noted to have sepsis, HIV infection, Type I diabetes or having both chronic joint pain and Type II diabetes were not used. The GTEx uniform processing pipeline provided relative abundance of genes in the form of normalized read counts as Transcripts per Million (TPM). GTEx RNA-seq assays used rRNA-depleted total RNA libraries containing reads from non-polyA transcripts with the proportion of such reads potentially varying between samples (Cui et al., 2010). We thus limited our analysis to validated coding genes by re-constraining the TPMs of coding genes [based on GENCODE annotation (Harrow et al., 2012)] to sum to a million.

### Separation Into Cohorts

We mined the associated clinical information for samples to classify donors into three cohorts based on well-understood phenotypic changes in peripheral nerves: those noted to have arthritis or rheumatoid arthritis (chronic joint pain cohort, CJP), those noted to suffer from Type II diabetes (Type II diabetes cohort, T2D, which often causes diabetic neuropathic pain), and those without either of these diseases (baseline cohort, BSL) based on studies in the literature that suggested phenotypic and molecular profile changes in peripheral nerves in arthritic and diabetic patients (Hur et al., 2011; Pongratz and Straub, 2013). We then studied whether these cohorts could be analyzed together, or needed to be analyzed separately.

**Figure 1:**
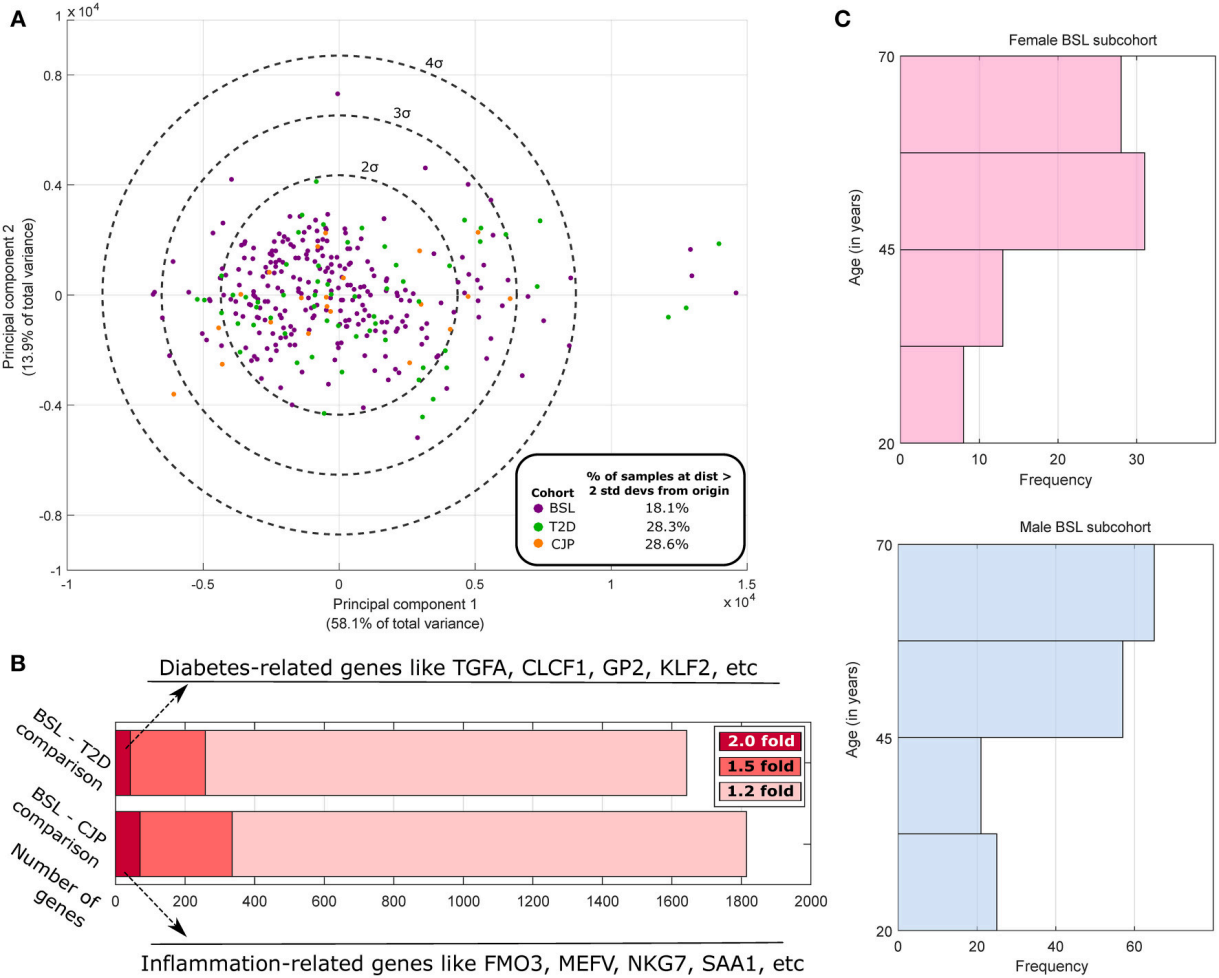
Population analysis of GTEx human tibial nerve samples. **(A)** PCA of the gene expression profile across all three cohorts of highly variable genes and subsequent plotting of the top 2 principal components show a larger fraction of outlier samples in CJP and T2D with respect to BSL. **(B)** The number of genes that are DE at 1.2-fold, 1.5-fold, and 2-fold when comparing the BSL with CJP and T2D are shown, along with some of the strongest DE genes. **(C)** The histogram of age in male and female BSL subcohorts show a similar distribution.

For the entire dataset including all three cohorts, only stably expressed genes (filtering out genes with median TPM across cohorts <0.5 or maximum TPM across cohorts <1.0) were analyzed, and each gene's normalized entropy score e_i_ (based on Ray et al., 2018) using samples from all cohorts was calculated as a measure of its variability:

$$\begin{array}{l} e_{i}=-\frac{1}{log_{2}\left(N\right)}\underset{j}{\sum }\frac{t_{i,j}}{\sum_{k}t_{i},k}log_{2}\frac{t_{i,j}}{\sum_{k}t_{i},k}\text{.} \end{array}$$

where *t*_*i, j*_ is the TPM of the *i*th gene in the *j*th sample, with i indexing genes and *j* and *k* indexing samples, *N* being the total number of samples, and log 0 being defined as 0. Principal Components Analysis (PCA) was performed for samples (Figure 1A) using stably expressed, highly variable genes (with normalized entropy using samples in all three cohorts in the 90th percentile or above). The top two principal components were found to account for 72% of the total variance in the dataset but did not spatially segregate the samples on the basis of sex or membership in the three cohorts, which is expected since sex differences or disease pathologies like arthritis or diabetes are not expected to cause transcriptome-wide changes (like cancer). Based on the first two PCA dimensions, we performed outlier analysis to identify samples that were two standard deviations or further away from the origin (around which the principal component values are centered). We note an ~50% increase in the proportion of outlier samples in the CJP and T2D cohorts (both >28%) as compared to the BSL cohort (18.1%). Additionally, a preliminary analysis finds that over 250 genes are differentially expressed at the effect size of 1.5-fold change or above for both BSL–CJP and BSL–T2D comparisons (Figure 1B). These include genes well-known to be implicated in inflammation (for BSL–CJP comparison) and diabetes (for BSL–T2D comparison). These findings suggest very distinct phenotypes in the three cohorts, and any sex difference studies in the cohorts are thus more suited for analysis separately due to the large inter-cohort differences.

However, while rigorous statistical hypotheses testing to identify DE genes was performed on BSL (168:80 Male: Female Ratio/MFR) to characterize sex differences in healthy hTN (Supplementary Table 1), hypotheses testing was not performed in CJP (11:10 MFR) or T2D (44:16 MFR) due to these cohorts being underpowered for DE gene identification. Expression values, median fold change and Strictly Standardized Mean Differences for male–female gene expression changes in the two cohorts are shown in Supplementary Tables 2, 3 as a starting point for future studies. Strictly standardized mean difference (*s*_*i*_) (Zhang, 2010) is a sample statistic that is often used to look at differences in effect size, while controlling for dispersion of data, under limited replication.

$$\begin{array}{l} s_{i}=\frac{\mu_{F,i}-\mu_{M,i}+\in }{\sqrt{\sigma_{F,i}^{2}+\sigma_{M,i}^{2}}+\in } \end{array}$$

where μ_*F, i*_ and μ_*M, i*_ are mean TPMs for female and male member of a cohort for the *i*th gene, σ_*F, i*_ and σ_*M, I*_ are the corresponding standard deviations with covariance assumed to be 0, with i indexing genes, and ε being a small smoothing factor (0.001) added to both the numerator and denominator.

### Analysis of Potential Confounding Factors in Male and Female BSL Subcohorts

Based on donor level data, we performed a thorough analysis of documented variables that could potentially be a confounding factor in our analysis (results shown in Table 1). We identified several variables that were potentially confounding factors, including demographic (age, race), anatomical (BMI), and medical history based (presence of autoimmune disease like lupus or scleroderma, and inflammatory diseases like sarcoidosis or cellulites) variables. We find no appreciable difference in the breakup of BSL male and female subcohorts by race, and distribution of age (Figure 1C) and BMI are also comparable. Examined disease conditions were either completely absent in both subcohorts, or present in a small number of males (sarcoidosis). Height and weight distributions for the male and female subcohorts are noted to be different, as expected.

**Table 1:** Statistics for BSL cohort.

	**Male**	**Female**
Cohort size	168	80
**CONSENT VARIABLES**		
**Consent group ( phv00169057.v7.p2.c1 )**		
General research use	100.0%	100.0%
No consent available	0.0%	0.0%
**VARIABLES DEFINING COHORT**		
Male	100.0%	0.0%
Female	0.0%	100.0%
**Type I/II diabetes (MHT1D or MHT2D)**		
Yes	0.0%	0.0%
No	100.0%	100.0%
**Arthritis (MHARTHTS or MHRA)**		
Yes	0.0%	0.0%
No	100.0%	100.0%
**Sepsis (MHSEPSIS)**		
Yes	0.0%	0.0%
No	100.0%	100.0%
**HIV serology testing outcome (LBHIV1NT)**		
Positive	0.0%	0.0%
Negative	94.6%	97.5%
Not performed	5.4%	2.5%
**DEMOGRAPHIC VARIABLES**		
**Race (RACE)**		
Asian	2.4%	2.5%
African American/Black	8.9%	11.3%
White	86.3%	86.3%
Am. Indian/Alaska Native	0.0%	0.0%
Unknown	2.4%	0.0%
**Age in years (AGE)**		
Mean	50.9	52.0
Standard deviation	14.0	12.9
**BMI/HEIGHT/WEIGHT**		
**Body mass index (BMI)**		
Mean	27.2	27.3
Standard deviation	3.9	3.9
**Height in inches (HGHT)**		
Mean	69.8	64.8
Standard deviation	3.1	2.8
**Weight in pounds (WGHT)**		
Mean	188.8	163.6
Standard deviation	32.3	26.8
**POTENTIALLY CONFOUNDING CLINICAL VARIABLES**		
**Lupus (MHLUPUS)**		
Yes	0.0%	0.0%
No	100.0%	100.0%
**Cellulites (MHCLLULTS)**		
Yes	0.0%	0.0%
No	100.0%	100.0%
**Scleroderma (MHSCLRDRM)**		
Yes	0.0%	0.0%
No	100.0%	100.0%
**Sarcoidosis (MHSRCDSS)**		
Yes	1.2%	0.0%
No	98.8%	100.0%

*Sample Characteristics. Distribution of relevant demographic, anatomical, clinical and medical history variables for the male and female subcohorts in BSL show no significant differences for potentially confounding variables*.

### DE Gene Identification

We performed non-parametric statistical hypotheses testing to identify DE genes. To minimize effects of multiple testing, we filtered out lowly expressed or undetectable genes (based on the conservative filtering criterion of median gene TPM <0.5 or maximum gene TPM <1.0 in both males and females in a cohort). We also filtered out genes that were ubiquitously expressed in BSL by calculating the normalized entropy (defined previously) across all samples in the BSL cohort. Since higher normalized entropy signifies more ubiquitous expression, we retained only those expressed genes in our analysis whose normalized entropy was less than the 75th percentile of normalized entropies of expressed coding genes, thus performing gene filtering in a manner agnostic to the sex of the samples. Wilcoxon rank-sum test (Wilcoxon, 1945) was used to calculate *p*-values for differences in the male and female sub-cohorts in BSL for the median (50th percentile) and the upper quartile (75th percentile), which can be robustly estimated given the cohort size. To test for differences at the 50th percentile (median), the entire male and female BSL sub-cohorts were used for comparison. In order to identify differences at the 75th percentile (upper quartile), only values greater than or equal to the medians of the male and female BSL sub-cohorts were used. To account for multiple testing, the Benjamini–Hochberg Procedure (BHP) (Benjamini and Hochberg, 1995) was used for both the 50th and 75th percentile tests with a False Discovery Rate (FDR) threshold of 0.05 for both tests (suggesting a combined FDR of 0.1). Since test statistics for the two tests are well-correlated due to overlap of data, the empirical FDR is effectively in the range (0.05, 0.1).

### Age–TPM Correlational Analysis

In order to understand how age affects gene abundances on the sex differentially expressed gene set, we first performed PCA on the BSL samples using only the 149 sex-DE genes. Correlation [Pearsons' Correlational Coefficient R (Pearson, 1895)] was calculated between the principal components and age. We also calculated Pearson's R separately for the male and female subcohorts of BSL for the sex-DE genes. For the male subcohort, BHP was performed on the corresponding *p*-values to identify R > 0.20 or R < −0.20 as a statistically significant correlation. For the female subcohort, while the number of samples were less, and the number of comparisons also less (Y chromosomal genes not tested), we used the same thresholds for consistency.

The list of sex differentially expressed genes are present in Table 2, and a searchable public database with gene expression values for all genes, as well as all code and data for the analyses can be found online at the companion website https://www.utdallas.edu/bbs/painneurosciencelab/sensoryomics/sexdiffnerve/.

**Table 2:**
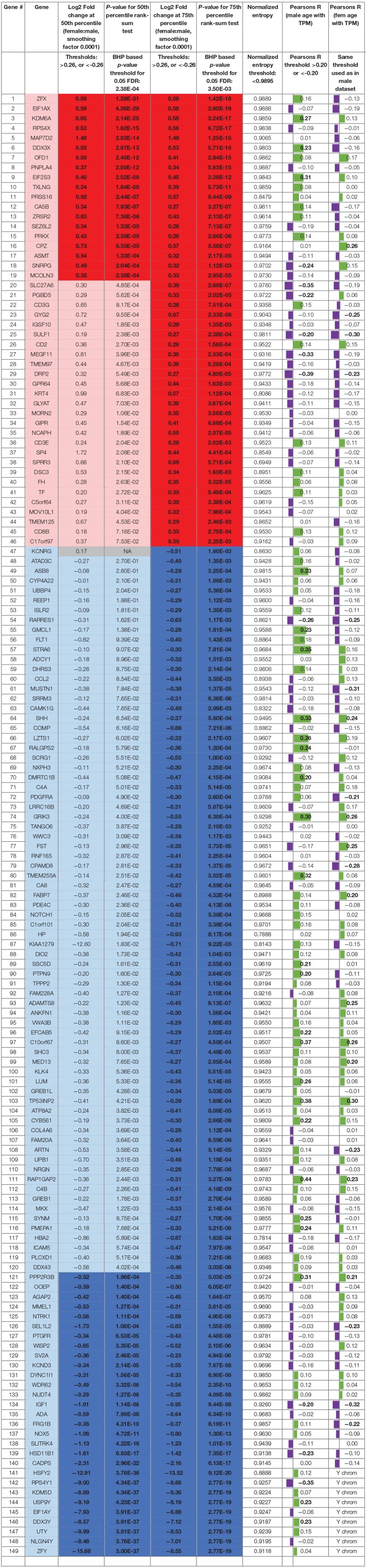
Statistically significant DE genes for BSL male vs. female subcohort analysis (statistically significant outcomes in boldface).

*Differentially expressed genes in human tibial nerve. Fold changes at the 50 and 75th percentile between males and females in BSL for 149 DE genes and p-values for Wilcoxon Rank Sum test are shown*.

*Dark red (DE in females) and dark blue boxes (DE in males) with boldface correspond to BHP-corrected statistically significant outcomes. Pearson's R for correlation with age in both male and female BSL subcohorts are shown with purple and green bars corresponding to negative and positive correlation with age, respectively, and boldface corresponding to effect sizes that are BHP-corrected statistically significant in males in BSL. Normalized entropy based on the BSL cohort values are shown*.

## Results

**Figure 2:**
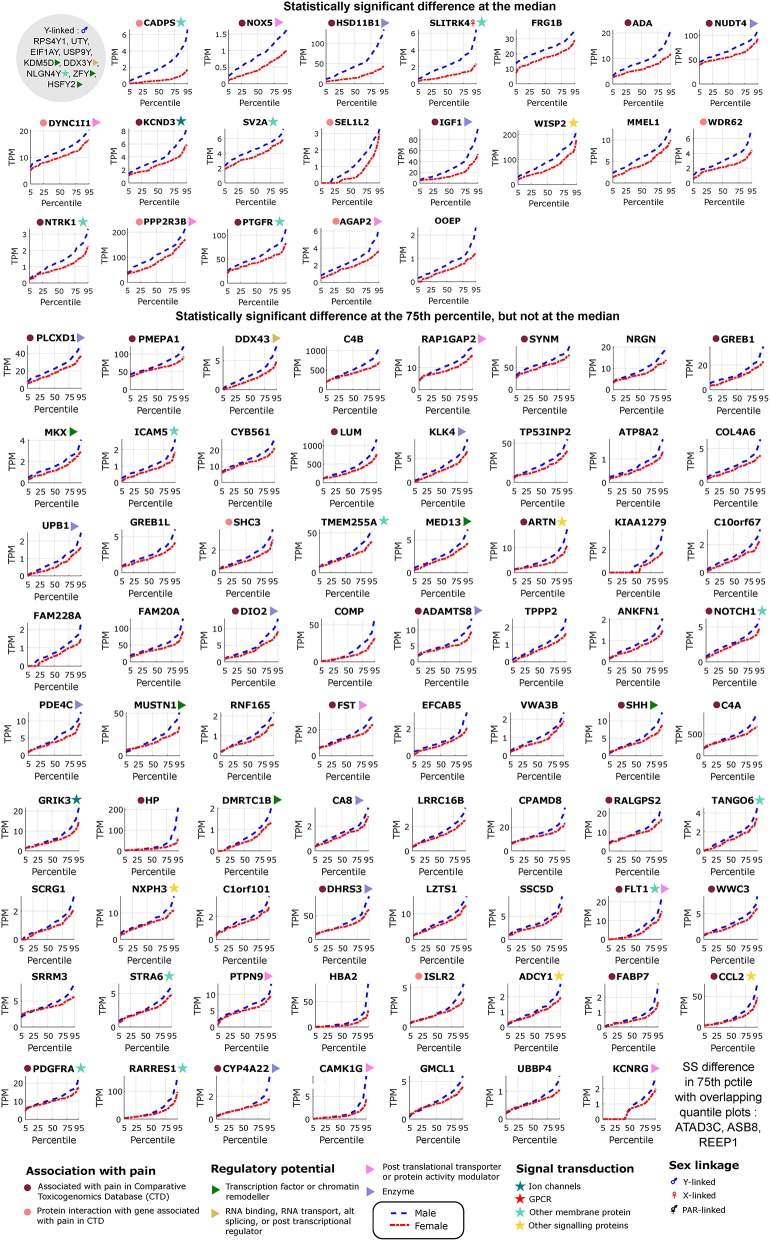
Differentially expressed genes in male tibial nerves. Quantile values for TPMs of sex-differentially expressed genes in BSL that are more abundant in males. Sex linkage, signal transduction and regulatory potential, and association with pain are also shown. Genes are shown in decreasing order of the area between the curves.

**Figure 3:**
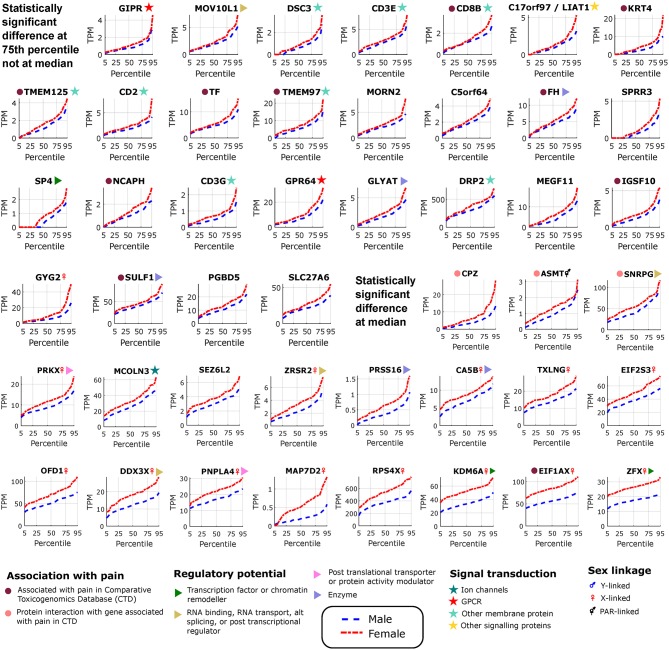
Differentially expressed genes in female tibial nerves. Quantile values for TPMs of sex-differentially expressed genes in BSL that are more abundant in females. Sex linkage, signal transduction and regulatory potential, and association with pain are also shown. Genes are shown in increasing order of the area between the curves.

We identified 29 genes in males, and 19 in females that were statistically significantly DE at ≥1.2-fold change (in their relative abundance/TPM) between the sexes in BSL at the 50th percentile. Additionally, we identified an additional 74 genes in males (Figure 2; Table 2), and 27 genes in females that were DE at the 75th percentile (Figure 3; Table 2). While several of these genes (including the 24 DE sex chromosomal genes) have been previously identified in the literature (Chen et al., 2016; Gershoni and Pietrokovski, 2017), our analysis identified new DE genes not identified in previous studies (Chen et al., 2016; Gershoni and Pietrokovski, 2017) including several that are potentially relevant to PNS function and pain mechanisms, including ISLR2, SP4, and TPPP2 (Chu et al., 2011; Aoki et al., 2014; Panza et al., 2015).

### Neural Tissue Enriched Genes

We found several genes that are sex-differential in expression and are enriched in expression in neural tissues. To identify neural tissue enriched genes, we identified genes in our DE gene list with high (≥0.5) Neural Proportion Scores (quantified in Ray et al., 2018) suggesting that they are primarily expressed in neurons or glia (in the CNS and PNS). NTRK1, which is known to be enriched in mammalian sensory neurons, was more abundant in males at both the 50th and 75th percentiles (Figure 2), suggesting sex-differential axonal mRNA trafficking. RNF165, involved in axonal growth, was also more abundant in male samples at the 75th percentile (Figure 2), and is another putative axonally transported mRNA. Well-known Schwann cell genes like Sonic Hedgehog (SHH) (Babcock et al., 2011) and Artemin (ARTN) (Lippoldt et al., 2013), that have been shown to be involved in pain, were also DE and higher in males at the 75th percentile (Figure 2). ISLR2 and MEGF11, known to be involved in axonal pathfinding, were differentially expressed in subsets of males and females, respectively (Figures 2, 3), and are potentially expressed in neurons and glia, respectively. Other neurally enriched genes that were DE in our datasets include PGBD5 and RNA helicase MOV10L1 in females [whose ortholog MOV10 has been shown to be implicated in nerve injury response in rodents (Melemedjian et al., 2011)], and AGAP2, NXPH3, and NRGN in males (Table 2). A small number of genes (SHH, *EFCAB5, FABP7*), which were neural tissue enriched, showed stronger or comparable sex-dependent expression in the hTN with respect to other tissues we examined (Supplementary Table 4).

**Figure 4:**
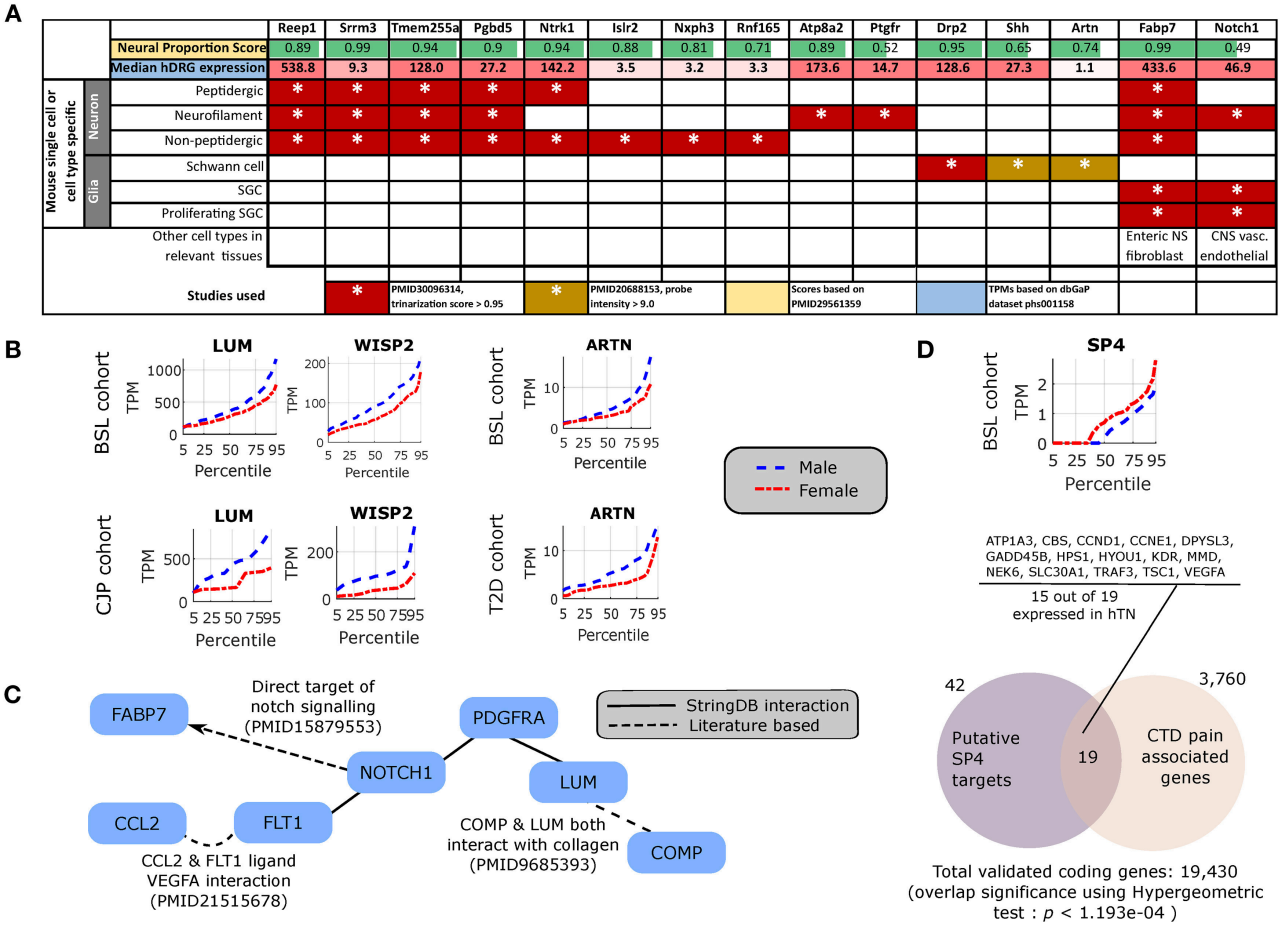
Cellular enrichment and functional roles of differentially expressed genes. **(A)** For DE genes that are expressed in the human DRG, enriched in neural tissues, and detected in murine PNS single cell RNA-sequencing, median expression in TPMs in the human DRG, enrichment of expression in human neural tissues [based on the human neural proportion score (Ray et al., 2018)], and murine cell types of expression in the DRG (based on Zeisel et al., 2018) are shown. **(B)** LUM and WISP2 show similar sex-differential expression trends in BSL and CJP, and ARTN shows similar trends in BSL and T2D. **(C)** Protein Interaction Network for pro-inflammatory genes that are more abundant in the male cohort, suggesting a central role of NOTCH1. **(D)** Quantile plot for SP4 male and female sub-cohorts, and gene set enrichment analysis showing overlap of known SP4 targets from TRANSFAC (Matys et al., 2006) expressed in the hTN and present in Comparative Toxicogenomics Database (Davis et al., 2012) based pain associated gene list.

To potentially identify the cell type of expression of neural tissue enriched genes, we analyzed the mouse nervous system single cell gene expression database at mousebrain.org (Zeisel et al., 2018) as well as the literature. Despite species' divergence of transcriptional regulation, we identified 15 genes whose murine orthologs were distinctly expressed in neuronal or glial cells in the mouse PNS (Figure 4A). Several of the neuronally expressed genes (NTRK1, ATP8A2, SRRM3, TMEM255A, RNF165, ISLR2) are expressed in the human DRG and have been shown in the literature to be important for axonal viability and routing or neurite outgrowth, suggesting that these are likely candidates for axonal mRNA transport and local translation.

### Pro-inflammatory Gene Signatures

The DE gene set with higher expression in subsets of male BSL sub-cohort showed a different pro-inflammatory gene signature (ARTN, HP, NOTCH1, CCL2, DIO2, and others), with respect to the female BSL sub-cohort (SULF1, GPR64, KRT4), suggesting sex-differential expression patterns of inflammatory gene markers under normal conditions (Table 2). This agrees with studies showing sex differences in clinical markers of inflammation (Casimir et al., 2010). Interestingly, the ratio of DE pro-inflammatory to anti-inflammatory genes in males were higher than in females, also in agreement with studies on human endothelial cells showing greater pro-inflammatory effects of androgens over estrogens (Annibalini et al., 2014). We analyzed the BSL male sub-cohort pro-inflammatory genes in T2D and CJP, and found only 3 inflammatory genes (LUM, WISP2, ARTN) with sex-dimorphic expression at comparable effect sizes in either T2D or CJP with respect to BSL (Figure 4B) suggesting that the pro-inflammatory gene signature is unlikely to be caused solely by a subset of donors in BSL that are suffering from inflammatory conditions unreported in the clinical record. The larger number of genes detected as more abundant in the male BSL sub-cohort can potentially be attributed to a combination of Y-chromosomal gene expression and downstream regulatory effects, and a larger set of DE pro-inflammatory genes.

We mined the set of pro-inflammatory protein that were more abundant in males for protein interaction based on both the literature and the StringDB database (Szklarczyk et al., 2016). We identified a Protein Interaction Network (PIN) connected through NOTCH1 (Figure 4C), known to be involved in neuropathic pain (Xie et al., 2015). This suggests that some of the pro-inflammatory genes more abundant in males (FABP7, FLT1, CCL2, PDGFRA, LUM, COMP) are potentially transcriptionally regulated by Notch signaling, or are co-regulated with NOTCH1. This is consistent with our knowledge of Notch signaling which is understood to drive a pro-inflammatory phenotype (Quillard and Charreau, 2013) in multiple tissues.

### DE Regulatory Genes

Underlying causes for such sex-differential gene expression include Y chromosome gene expression, incomplete X inactivation in females (Gershoni and Pietrokovski, 2017), differential androgen and estrogen receptor driven regulation between sexes (Chen et al., 2016) and transcription regulatory programs controlled directly or indirectly by sex chromosomal gene products. Additionally, we find DE transcriptional regulators on autosomes including DMRTC1B and MED13 in males, and SP4 in females. SP4 is known to regulate pain-related genes, including several expressed in hTN (Figure 4D). While fold changes in these autosomal genes under baseline conditions are unlikely to affect transcription in their regulatory targets, the presence of sex-differential expression in a subset of samples suggests that under pathological conditions like inflammation and pain, more prominent sex-differential expression of these transcriptional regulators could potentially drive sex-specific regulatory programs.

### Correlation of Gene Abundance With Age

**Figure 5:**
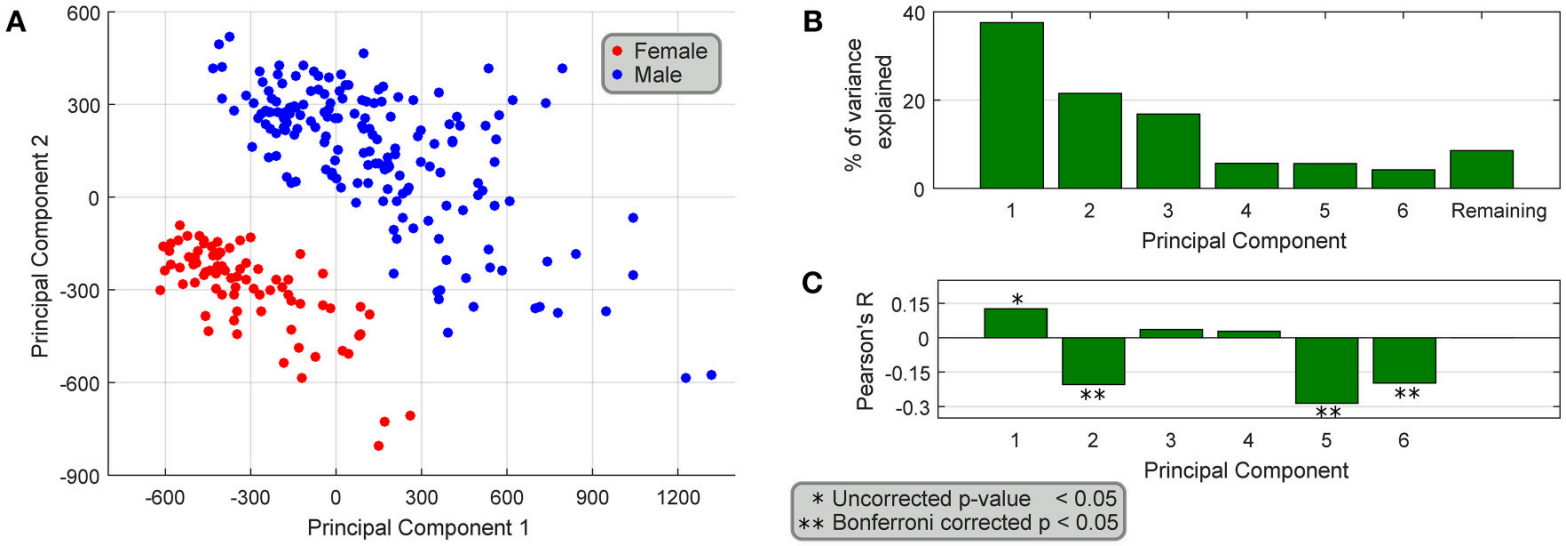
Association of differentially expressed genes with age. **(A)** PCA of the 149 sex-differentially expressed genes in BSL yields top two principal components that spatially segregate male and female samples. **(B)** The top six principal components explain >90% of the variance, and **(C)** 4 out of the top 6 principal components are weakly correlated with age of the samples.

We performed a principal components analysis of only the 149 sex differentially expressed genes, with the top two components clearly spatially separating male and female samples (Figure 5A). We also find that the top six principal components accounted for >90% of the variance (Figure 5B), but more interestingly four of the top six components are correlated with age (Figure 5C), both positively and negatively, showing that different parts of sex differential gene expression are both positively and negatively correlated with age. At the level of individual genes, we find that several genes have very different (weak) correlational patterns in males and females (Table 2). One possible explanation for this is regulation (direct or indirect) by sex hormones, especially via ESRA and ESRB, which have been studied in GTEx datasets (Chen et al., 2016). In fact, we find genes in our datasets (DDX3X, SULF1, TMEM125, and others more abundant in females; FLT1, MUSTN1, COMP, ARTN, REEP1, SEL1L2, and others more abundant in males) with negative correlation between female BSL gene abundance values and age, and a smaller correlative effect (or positive correlation) in male BSL samples. Several genes in our dataset like MUSTN1, REEP1, and FLT1 have been shown to be regulated by estrogen in the literature.

### Potential Protein Interaction Networks (PINs)

**Figure 6:**
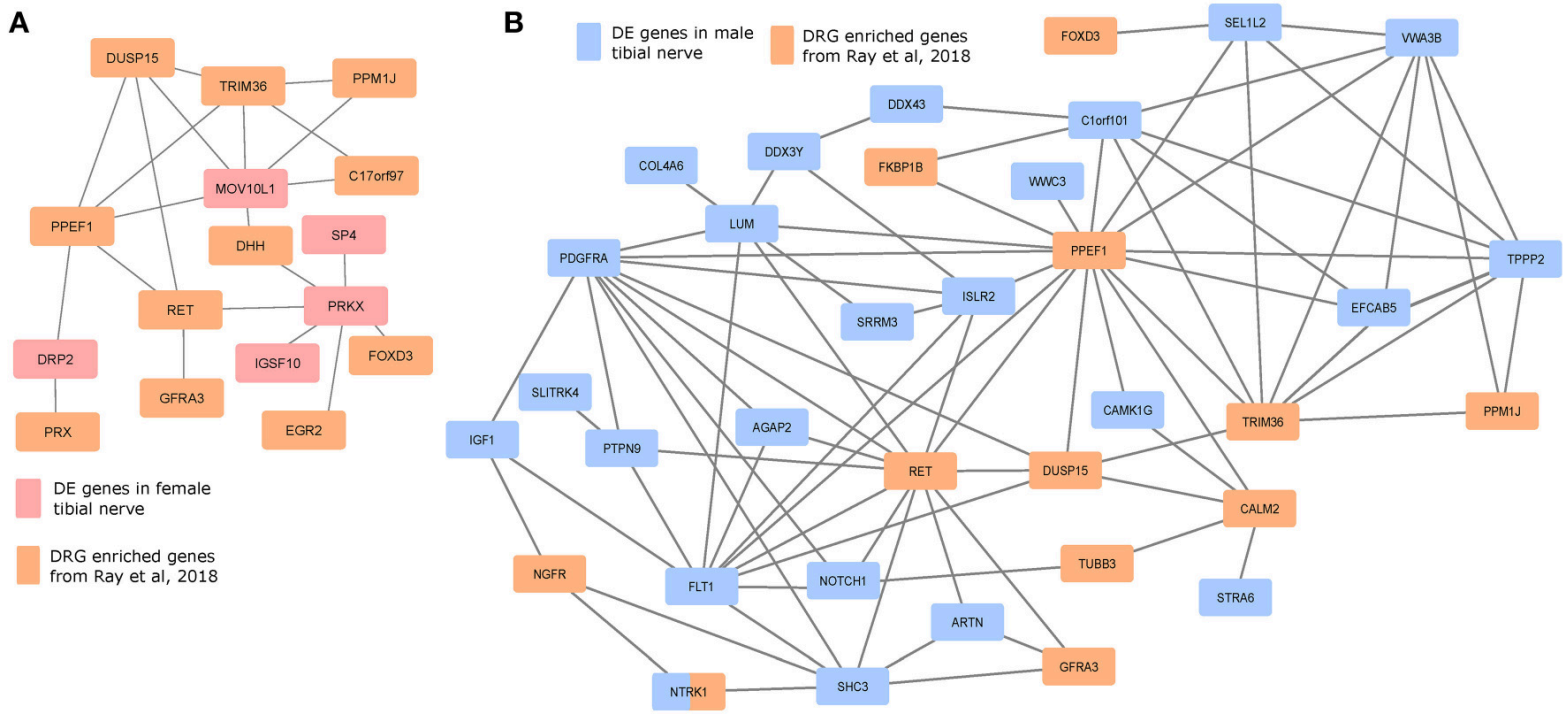
Protein interaction networks of sex differentially expressed genes and human DRG enriched genes. Largest connected components of PINs based on known StringDB interactions between mammalian DRG-enriched genes (based on Ray et al., 2018), and DE genes that are more abundant in the **(A)** female BSL sub-cohort, and **(B)** male sub-cohort.

We investigated whether the gene products of the DE gene sets potentially interacted with genes whose expression is enriched in mammalian DRGs, which would help identify candidate sex-differential PINs in the PNS (Figures 6A,B). We used DRG-enriched genes from Ray et al. (2018) and our DE gene sets to identify putative PINs. The largest connected components from PINs generated using the StringDB database (Szklarczyk et al., 2016) show multiple DRG-enriched and male-preferentially expressed genes known to be expressed in glia (DUSP15, PRX, EGR2, DHH, FOXD3, ARTN), and involved in pain and inflammation, which points to a potential role for glia in sex differential pain processing in human peripheral nerves [shown in preclinical models (Averitt et al., 2018)]. Additionally, the presence of several neuronally expressed genes in the PIN among the set of DRG-enriched genes (GFRA3, NTRK1, NGFR, RET, and PPM1J) also suggests sex-differential glia-neuron crosstalk, which in turn can affect neuronal plasticity and excitability differently between the sexes.

### Roles in Pain and Neuro-Immunity

Out of 149 DE genes we identified, 56 (>33%) genes have known roles in pain, inflammation, innate immunity and other neuro-immune functions (Table 3). Sex differences in mammalian neuro-immune systems have been studied and linked to disease prevalence and susceptibility (Osborne et al., 2018), with PNS and CNS immune response sex differences studied in rodent pain models (Sorge et al., 2015; Mapplebeck et al., 2016; Lopes et al., 2017). In hTN, we find that DE genes involved in the innate immune system (C4A/B, CPAMD8 in males; DDX3X, TF in females) as well as genes known to be expressed in infiltrating or resident immune cells (ADA, PDGFRA in males; CD2, CD8B in females). We also found 34 genes that were associated with pain based on either the Comparative Toxicogenomics Database (Davis et al., 2012) or the Human Pain Genetics Database (Meloto et al., 2018), including genes that are central to pain pathways like NTRK1, and CCL2 in males and TMEM97 in females (Sahn et al., 2017; Ray et al., 2018).

**Table 3:**
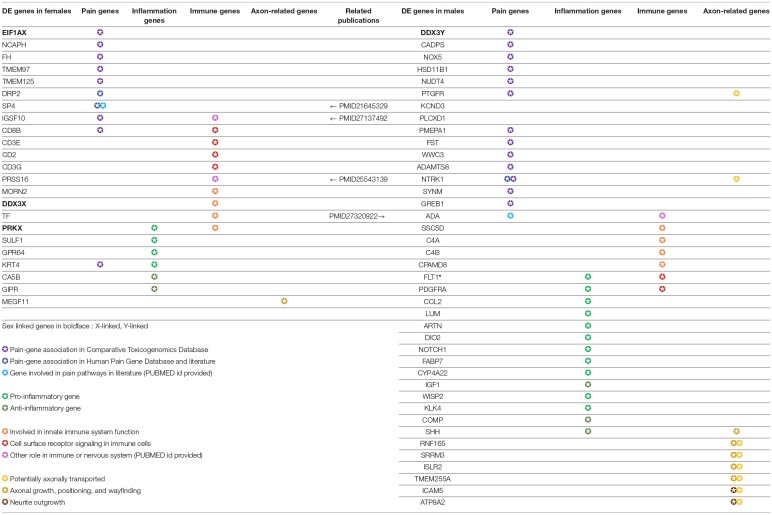
Functionally relevant genes that are sex-differentially expressed in the human tibial nerve.

^*^Primarily expressed in endothelial cells, also reported in immune cells.

*Functional annotation of sex differentially expressed genes. Functional annotations for sex-differentially expressed genes in BSL showing whether they are involved in pain, inflammation, immune system functions, axonal growth/neurite outgrowth or are potentially axonally transported, based on literature. ICAM5 is known to be present in dendrites, and has not been shown to be present in axons. Other potentially axonally transported transcripts have known roles in axonal viability, growth and wayfinding or neurite outgrowth*.

## Discussion

Our work provides a biostatistical framework, and thoroughly catalogs sex-differential gene expression in hTN. The public resource (https://www.utdallas.edu/bbs/painneurosciencelab/sensoryomics/sexdiffnerve/) we generated provides a starting point for sex difference studies in human peripheral nerve drug target discovery, gene regulation and pathophysiology. We cataloged sex differences in gene expression in the PNS finding that many sexually dimorphic genes have known associations with pain, inflammation and neuro-immune interactions. This resource can be used for hypothesis-driven work to identify cell type and sub-compartment specificity of gene expression (e.g., NTRK1, LUM) by *in situ* hybridization or other techniques. These types of experiments will be important in identifying whether neuron-specific transcripts found in the tibial nerve are *bona-fide* axonally-localized transcripts or if they represent low level expression in other cell types that have not been recognized in single cell datasets to date. For genes like NTRK1 we favor the hypothesis that this is an axonally localized mRNA. From that perspective, it is likewise interesting that some of these genes are DE suggesting that sex hormones may drive mRNA localization in neurons. We are not aware of any previous studies that describe sex hormone-mediated control of axonal mRNA localization.

Our study can also be used to back-translate into transgenic preclinical models to identify potential sex differences in mechanisms of PNS physiology that may be relevant to pain therapeutics. While studies of molecular mechanism in human cohorts are difficult, sex differences in regulatory programs, signaling cascades and pathophysiological and behavioral changes can be studied in rodent-based perturbation models. Both SP4 and NOTCH1 are known to play key regulatory roles with respect to gene products that drive pain pathways (Chu et al., 2011; Xie et al., 2015), and are good candidates for further study.

While there are no rodent studies of baseline transcriptome sex differences in peripheral nerves, O'Brien et al. (2016) found sex differences in transcriptomes of sciatic nerves of diabetic mouse models show clear differences in the inflammasome. It is possible that the baseline differences in molecular profiles that we identified drive greater sexual dimorphisms under neuropathic conditions. While our study was underpowered for identifying sex-DE genes in the CJP and T2D cohorts, it can be expanded as GTEx cohort sizes continue to grow to potentially identify clinically relevant sex differences in CJP or T2D. Moreover, co-expressed (and putatively co-regulated) gene modules based on Whole Genome Correlation Network Analysis (Langfelder and Horvath, 2008) can be identified by finding correlated expression changes across cohorts and sexes. Given that sex differences in gene expression likely contribute to sexual dimorphism in neurological disease, such as chronic pain, exploiting these transcriptomic resources will be increasingly important for mechanism and drug discovery.

There are limitations to our work. As mentioned in the previous paragraph, our study was not able to identify sex-differential expression in Type II diabetes or arthritis, largely due to the small sample size. However, our work does identify some putative targets for future prospective studies. Most prominently, our findings suggest differential expression of immune-regulatory genes in tibial nerve of males and females. It may be reasonable to hypothesize that these differences will become more pronounced in chronic pain disorders. It is striking that we observed higher expression of some macrophage-expressed and/or macrophage recruiting genes (CCL2, NOTCH1) at higher levels in males given that many recent studies have identified macrophages and microglia as important contributors to pain specifically in male rodents (Mapplebeck et al., 2016; Lopes et al., 2017; Averitt et al., 2018; Inyang et al., 2018). We also observed higher expression of a well-known T cell gene in female TN, CD8B. This is interesting because T cells have been associated both with pain promotion (Sorge et al., 2015) and pain resolution (Krukowski et al., 2016) in female mice. Another shortcoming is that we have not addressed any specific hypotheses with interventional, prospective studies. This will be a future goal using rodent models. Finally, there is a potential confound of age in our analysis of sexual dimorphisms in gene expression. Our detailed analysis of this confound shows that age cannot explain all of the differences observed, and even when it does, it may be explained by changes in sex hormones across the lifespan.

In conclusion, our work demonstrates robust sexual dimorphisms in gene expression in the human tibial nerve. Some of these dimorphisms are likely important for understanding how the immune system interacts with the peripheral nervous system. Insofar as this is a major focus of current research into acute and chronic pain mechanisms (Mapplebeck et al., 2016; Lopes et al., 2017; Averitt et al., 2018; Inyang et al., 2018), these findings give new insight into translating findings from animal models into humans, as well as potential back-translation for testing of new hypotheses in animal models based on human molecular data.

## Author Contributions

PR, AA, GD, and TP conceived the project. PR designed and performed experiments, analyzed and interpreted the data. JK performed exon-level analysis. DT-F performed pilot experiments. AW performed experiments for Supplementary Table 4. PR drew all figures. PR and TP wrote the paper, with input from GD.

### Conflict of Interest Statement

The authors declare that the research was conducted in the absence of any commercial or financial relationships that could be construed as a potential conflict of interest.

## Abbreviations

hTN: human tibial nerve
CJP: chronic joint pain cohort
T2D: Type II diabetes cohort
BSL: baseline cohort
DE: differentially expressed
BHP: Benjamini-Hochberg procedure
MFR: male:female ratio
PIN: protein interaction network
DRG: dorsal root ganglion
PNS: peripheral nervous system
PAR: pseudo-autosomal region
TPM: transcripts per million
BMI: body mass index
PCA: principal components analysis.
