# Erratum: Transcriptome Analysis of the Human Tibial Nerve Identifies Sexually Dimorphic Expression of Genes Involved in Pain, Inflammation, and Neuro-Immunity

**DOI:** 10.3389/fnmol.2019.00127

**Published:** 2019-05-22

**Authors:** Frontiers Production Office

**Affiliations:** Frontiers Production Office, Frontiers Media SA, Lausanne, Switzerland

**Keywords:** sex-differential gene expression, human peripheral nerve transcriptome, peripheral nervous system sex differences, pain genes, pro-inflammatory genes

Due to a production error, the wrong file was published as Supplementary Table 2. The original article has been updated with the correct Supplementary Table 2. The publisher apologizes for this mistake.

